# Clinical Features and Prognostic Significance of *NOTCH1* Mutations in Diffuse Large B-Cell Lymphoma

**DOI:** 10.3389/fonc.2021.746577

**Published:** 2021-12-09

**Authors:** Zhongqi Li, Fang Yu, Wenle Ye, Liping Mao, Jiansong Huang, Yang Shao, Junrong Yan, Wenjuan Yu, Jie Jin, Jinghan Wang

**Affiliations:** ^1^ The Department of Surgical Oncology, The First Affiliated Hospital, Zhejiang University, Hangzhou, China; ^2^ Department of Pathology, The First Affiliated Hospital of Zhejiang University, Hangzhou, China; ^3^ Department of Hematology, The First Affiliated Hospital, Zhejiang University College of Medicine, Hangzhou, China; ^4^ The First Affiliated Hospital of Zhejiang University, Key Laboratory of Hematologic Malignancies, Diagnosis and Treatment, Hangzhou, China; ^5^ Institute of Hematology, Zhejiang University, Hangzhou, China; ^6^ Cancer Center, Zhejiang University, Hangzhou, China; ^7^ Medical Department, Nanjing Geneseeq Technology Inc., Nanjing, China

**Keywords:** next generation sequencing (NGS), diffuse large B-cell lymphoma, clinical decision making, NOTCH1 mutations, clinical outcome

## Abstract

Diffuse large B-cell lymphoma (DLBCL) is a heterogeneous group of large lymphoid B cell malignancy with distinct clinical and genetic features. Recently, *NOTCH1* mutations were identified in DLBCL cases by Next-generation sequencing (NGS), but the clinical features and prognostic impact were not systematically studied. Here, *NOTCH1* genes in 161 DLBCL samples were sequenced by NGS. The prognostic value of *NOTCH1* mutations was assessed in the context of clinical and laboratory factors, such as international prognostic index (IPI), cell-of-origin classification, double expression of BCL2 and c-MYC. The combined data from three Western cohorts were used to validate these results. As a result, *NOTCH1* mutations were found in 17(10.6%) patients, and three patients had a hotspot mutation of c.7541_7542delCT. The presence of *NOTCH1* mutations was significantly associated with poor complete response and progression free survival(PFS), which was independent of established clinical and laboratory parameters. In addition, 30 (1.92%) of 1562 patients treated with R-CHOP regimen in those combined Western cohorts had *NOTCH1* mutations. Meta-analysis of the Western cohorts confirmed that *NOTCH1* mutations were also associated with poor PFS and OS. In conclusion, DLBCL patients with the *NOTCH1* mutations have worse PFS and OS, and the *NOTCH1* mutations can be used as an independent predictor for patients with DLBCL.

## Introduction

The NOTCH pathway is a highly conserved signaling pathway, which is widely involved in cellular proliferation, differentiation, and apoptosis (1). There are four types of Notch receptors in mammals, such as NOTCH1, NOTCH2, NOTCH3, and NOTCH4 proteins. NOTCH1 and NOTCH2 receptors are highly expressed in many tissues, while NOTCH3 is mainly seen in vascular smooth muscles, and NOTCH4 is usually observed in endothelium (2). Notably, most of the genetic changes of the Notch receptors were observed in the NOTCH1 gene (3–6). This receptor consists of an extracellular component, followed by a transmembrane domain and an intracellular region (NICD). There are at least two forms to activate the Notch-1 mediated signals: ligand-dependent and ligand-independent activation pathways, respectively (7). When the extracellular domain binds to its ligand, the ligand-dependent NOTCH 1 signaling is activated (8). While, gain-of-function mutations in *NOTCH1* gene often lead to the ligand-independent activation in pathological conditions (8). After activation, NICD is cleaved from the intracellular domain and then translocated into the nucleus, leading to the transcription of Notch target genes, including the MYC oncogene (5, 8).

The first report about NOTCH1 receptors in malignancies was the observation of a constitutive activation of NOTCH1 signals in T-cell acute lymphoblastic leukemia with a t(7;9)(q34;q34.3) chromosome translocation (9). Subsequently, more and more studies discovered activation of NOTCH1 receptor occurs in several solid tumors such as colorectal cancer (8), head and neck cancer (10), lung cancer (11), and melanoma (12), and other hematologic malignancies such as chronic lymphocytic leukemia (13, 14), mantle cell lymphoma (15), and Hodgkin’s lymphoma (5). Biologically, defects in the Notch signaling pathway would contribute to the development of congenital disorders, viral infections, and cancer (1–3). Clinically, patients with *NOTCH1* mutations are often associated with poorer clinical outcomes and a higher risk of disease progression (13, 16). In contrast, tumor suppressive effect was also reported in some studies. For example, *NOTCH1* deficiency in skin can lead to the development of skin tumors (17). Thus, the multifaceted role of *NOTCH1* signaling in cancer inhibition or promotion depends on the influence of cellular microenvironment (18).

Diffuse large B cell lymphoma (DLBCL) is the most common type of non-Hodgkin lymphoma and has received extensive attention in terms of genetic findings and clinical outcomes (19). Using whole genome/exome sequencing, plenty of mutations were found in DBLCL (6). However, the biological significance and clinical associations of each mutated gene still need further investigation. It has been reported that the *NOTCH1* mutations are associated with reduced benefit of anti-CD20 chemoimmunotherapy regimens in chronic lymphocytic leukemia (20), and its clinical significance in DLBCL is unclear (6, 21, 22). In this study, we enrolled a relatively large cohort of DLBCL patients to investigate the clinical and biological characteristics of NOTCH1 mutations in DLBCL patients.

## Materials and Methods

In this study, 161 newly diagnosed DLBCL patients were enrolled from 2013 to 2020 in the hematological department of our hospital. The pathological diagnoses of DLBCL was based on the World Health Organization Classification (23). We included DLBCL patients with fresh frozen tumor tissues, older than 18 years, and received R-CHOP(rituximab 375 mg/m2 on Day 0, cyclophosphamide 750 mg/m2, doxorubicin 50 mg/m2, and vincristine 1.4 mg/m2 on Day 1, and prednisone 50 mg/m2 orally on Days 1-5) chemotherapy. Patients with HIV infection, pregnancy, another cancer, and double and/or triple hits lymphoma were excluded in this study. Clinical and laboratory information were retrospectively collected from the medical records at the time of DLBCL diagnosis. The computed tomography (CT) scans and/or positron emission tomography-CT, and bone marrow biopsy were used to assess the treatment response, and disease progression. All of the subjects were well-informed about the study and provided written informed consent to participate in the study. The study was approved by the Institutional Review Board of our hospital (No : IIT20210369A).

### Immunohistochemistry and Fluorescent *In Situ* Hybridization Analyses

Formalin-fixed paraffin-embedded tissue sections were used for IHC and FISH analyses. Automated IHC for CD20, CD10, BCL2, BCL6, MUM1, c-MYC, Ki-67 were performed on 4-μm-think tissue sections using an automated slide stainer, the VentanaBenchmark XT (Ventana Medical Systems). Cases with more than 40% positive cells of MYC and 50% of BCL2 were identified as double expressor lymphoma(DEL). Bcl-2, Bcl-6 and c-Myc fracture probes were applied to the sections, and details of FISH methods were previously described (24). COO classification was determined by Hans’s algorithm (25).

### Targeted Next-Generation Sequencing

*NOTCH1* mutations were performed by the targeted NGS tests. Genomic DNA was extracted from the formalin-fixed paraffin-embedded tissue sections. The detailed methods were reported in supplementary methods. Mutation analyses of *NOTCH1* were carried out as described previously (26). The primers were depicted in **Table S1**.

### Statistical Analysis

Our major aim was to evaluate the prognostic significance of *NOTCH1* mutations on progression free survival (PFS) in DLBCL patients after RCHOP chemotherapy. PFS was defined as the time from disease diagnosis until the time of progression, relapse or death from any cause. Overall survival (OS) was defined as time from the date of diagnosis until death due to any cause or the last follow-up. Complete response (CR) was defined according to the Revised Response Criteria for Malignant Lymphoma (27). The log-rank test in the Kaplan-Meier survival model was used to evaluate the prognostic impact of categorical variables. Univariate and multivariate analyses with Cox proportional hazards models were performed to assess significant predictors. The proportional-hazards assumption was checked for each variable before fitting Cox models. The survival meta-analyses were conducted by the “meta” package (28), the detailed information about the mutation sites was illustrated by the “trackViewer” package (29). The median, interquartile range and frequency counts were used to summarize the distribution of clinical data. Fisher’s exact test and nonparameter T-test were used to test the categorical and continuous variables, respectively. All statistical analyses were conducted with R statistic packages, version 3.6.1 (www.r-project.org). The two-sided level of significance was set at p-value < 0.05.

## Results

### *NOTCH1* Mutations in DLBCL Patients

**Figure 1:**
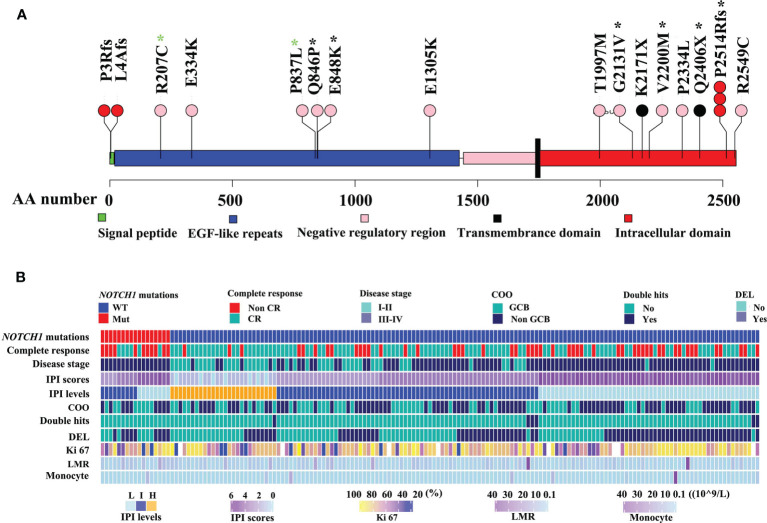
Mutation maps of the NOTCH1 protein **(A)**. The x-axis reports the amino acid(AA) number. The circles are colored with respect to the corresponding mutation types: “black” representing Non-sense mutations, “red” equaling to frameshift mutations, “pink” representing missense mutations. Black stars representing mutations identified by Sanger sequencing, while green stars representing no mutations identified by Sanger sequencing. The detailed clinical information of DLBCL was illustrated **(B)**. IPI, International Prognostic Index; non-GCB, non-germinal center B-cell-like lymphoma; DEL, double expressor lymphoma; HB, hemoglobin; LMR, lymphoma-to-monocyte ratio.

As illustrated in **Figure 1**, *NOTCH1* mutations were detected in 17 of 161 DLBCL patients (10.6%), specifically, including one splice mutation, two non-sense mutations, five frame shift mutations, and eleven missense mutations (**Figure 1A** and **Table S2**). We conducted the Sanger Sequencing to examine 8 out of 16 mutated sites of NOTCH1, and validated 2 mutated sites in extracellular regions such as c.2537 A>C (p.Q846P) and c.2542G>A (p.E848K), and four sites in intracellular domains like c.6392G>T (p.G2131V), c.6598G>A (p.V2200M), c.7541_7542delCT(p.P2514Rfs) and c.7216C>T (p.Q2406*). Two sites (R207C and P837L) in the EGF-like repeats regions were not validated by the Sanger Sequencing probably due to the relatively low tumor alleles. The details of Sanger sequencing were depicted in the **Figure S1** and **Table S2**. Generally, splice, framing and non-sense mutations often lead to large-scale changes in proteins. However, missense mutations lead to the substitution of different amino acids, which in turn have different effects on the protein’s function. Therefore, we further estimated the effects of missense mutations on protein function by using the PANTHER cSNP tool (30). There results showed that all of the missense mutations may impair the function of NOTCH1 protein, among which the highest score were G2131V, E334K, and V2200M missense mutations, implying the more likely deleterious effect on proteins (**Figure S2**). *NOTCH1* missense mutations and the recurrent c.7541_7542delCT (validated by Sanger sequencing in **Figure S1**) are supposed to affect the NOTCH activity. However, the non-sense mutations and the frameshift deletion in the initial region of *NOTCH1* gene probably lead to the absence of protein expression. Thus, we named mutations potentially affecting the NOCH1 activity as type 1 group and mutations probably leading to the absence of protein expression as type 2 group. In this study, there were no differences in their relationship with the clinical parameters (**Table S3**).

### Clinical Characteristics of DLBCL Patients With *NOTCH1* Mutations

Clinical features of DLBCL patients with *NOTCH1* mutations are summarized in **Table S4**. Patients with *NOTCH1* mutations were predominated in stage III-IV(P=0.003, **Figure 1B**). *NOTCH1* mutations were significantly associated with lower blood monocyte counts (P=0.031) but higher lymphoma/monocyte ratio (P=0.02) and higher hemoglobin levels (P=0.006). Notably, patients with the *NOTCH1* mutations had a lower complete response rate (P=0.028) than those without *NOTCH1* mutations. There was no statistically significant correlation between *NOTCH1* mutations and gender, age, international prognostic index (IPI), cell-of-origin (COO) classification, double expressor lymphoma (DEL), white blood cell count (WBC), platelet count, neutrophil counts and other variables (**Table S4**).

### Prediction of *NOCH1* Mutations in DLBCL

**Figure 2:**
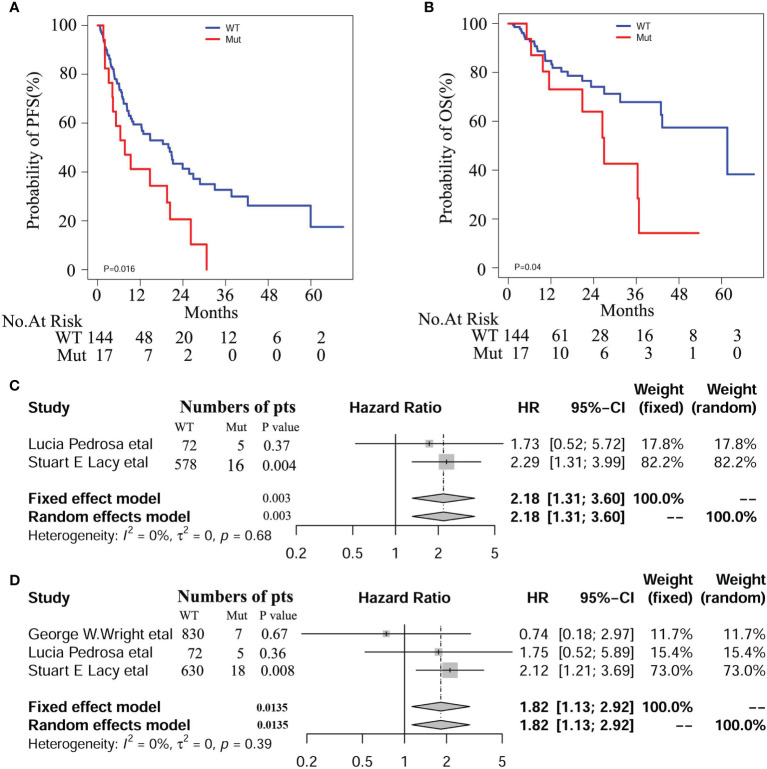
Survival curves of PFS **(A)** and OS **(B)** in our DLBCL patients with and with *NOTCH1* mutations. Meta-analyses of PFS **(C)** and OS **(D)** in the Western cohorts of DLBCL patients.

At the median follow-up of 43.3 months, 3-year progression-free survival (PFS) and overall survival (OS) rates for DLBCL patients were 28% and 60%, respectively. In this study, we also evaluated the influence of recognized prognostic factors such as IPI, COO and DEL classifications on prognosis (**Figures S3–S5** and **Table S5**). Consistent with other studies, higher levels of IPI, non-GCB and DEL predicted shorter PFS and OS, respectively. Additionally, hemoglobin (HB) and lymphoma/monocyte ratio(LMR) also have some prognostic values for PFS or OS. Notably, there was a significant difference in PFS and OS between patients with and without *NOTCH1* mutations in our DLBCL patients (**Figures 2A, B**). In multivariate analyses, the effect of *NOTCH1* mutations on poor PFS[HR(95% CI), 2.373(1.296,4.344); P=0.005] and OS [HR(95% CI), 5.025(2.001,12.62); P<0.001] persisted, and its prognostic impact was independent of GCB subtypes and/or non-DEL in DLPCL patients (**Table 1**). In addition, we performed a multivariate analysis of *NOTCH1* mutations and treatment response. Similarly, *NOTCH1* mutations were inversely and independently associated with complete remission after chemotherapy (**Figure S6**).

**Table 1:** Multivariable analyses of PFS and OS in DLBCL patients.

Variables	Progression free survival		Overall survival	
	P values	HR(95%CI)	P values	HR(95%CI)
Mutant vs. WT	0.005	2.373 (1.296,4.344)	<0.001	5.025 (2.001,12.62)
IPI scores	<0.001	1.688 (1.380,2.064)	<0.001	1.964 (1.361,2.833)
Non-GCB vs. GCB	0.244	1.354 (0.813,2.253)	0.030	2.661 (1.125,6.295)
DEL vs Non-DEL	0.979	0.993 (0.576,1.711)	0.720	1.197 (0.452,3.173)
LMR	0.429	0.824 (0.511,1.330)	0.010	0.369 (0.170,0.800)
HB	0.146	0.992 (0.980,1.003)	0.200	0.989 (0.974,1.006)
KI67	0.656	1.003 (0.991,1.014)	0.290	1.012 (0.990,1.034)

IPI, International Prognostic Index; Non-GCB, Non-germinal center B-cell-like lymphoma; DEL, double expressor lymphoma; HB, hemoglobin; LMR, lymphoma-to-monocyte ratio.

### Meta-Analyses of *NOTCH1* Mutations in Western Cohorts

We enrolled 1562 patients with DLBCL treated with R-CHOP regimen, including 837 from George W. Wright et al. (22), 77 from Lucía Pedrosa et al. (31), and 648 from Stuart E. Lacy and colleagues (32), respectively. Among these patients, 30 (1.92%) cases were identified as *NOTCH1* mutations. Detailed mutation information is illustrated in **Figure S7**, including frame shift mutation, non-sense mutation, splicing site mutation, and missense mutation. We conducted a meta-analysis on these three cohorts and found a significant correlation between *NOTCH1* mutations and PFS (HR 95%(CI), 2.18 [1.31; 3.60]; P=0.0025), and OS (HR 95%(CI), 1.82 [1.13; 2.92]; P=0.014, **Figure 2**). Besides, we combined the individual data of the three cohorts to obtain similar results. As shown in the **Figure S8**, there was a significant correlation between *NOTCH1* mutations and PFS (P=0.005 and OS(P=0.02), respectively.

## Discussion

Whole genome and exome sequencings have revealed numerous somatic mutations that occur repeatedly in DLBCL. A systematic and in-depth study of these mutant genes can help us better screen out high-risk cases and predict new therapeutic targets for DLBCL. In this study, we included a relatively large cohort of DLBCL patients, analyzed *NOTCH1* gene mutations by NGS sequencing, and evaluated the prognostic value of *NOTCH1* mutations and other recognized clinical and laboratory risk stratification factors. Finally, we performed a meta-analysis on three published Western cohorts to verify our findings.

As a result, *NOTCH1* mutations were found in 17(10.6%) patients, and three patients had a hotspot mutation of c.7541_7542delCT. In comparison, the frequency of *NOTCH1* mutations in 1562 Western patients treated with R-CHOP was just 1.92%. *NOTCH1* mutations are more common in the extracellular regions in the Chinese patients. Additionally, most mutated sites in the intracellular domains are different between the Chinese and Western patients. In order to confirm these new mutations in the Chinese patients, we conducted the Sanger Sequencing. In this study, we found 2 mutated sites in extracellular regions such as c.2537 A>C and c.2542G>A, and four mutated sites like c.6392G>T, c.6598G>A, c.7541_7542delCT, and c.7216C>T in the intracellular domains. Due to no high quality samples and PCR failure, we cannot validate the other mutation sites by Sanger sequencing, particularly for two mutations in the signal peptide. Different technology platforms, analysis pipelines and statistical methods may be one of the main reasons for the differences. For example, Noel F. C. C. de Miranda et al. used Sanger sequencing to detect only hotspots mutations(p.1500-1800 and p.2300-2555), 6% of DLBCL samples were identified (33). Another possible reason may be the difference in target populations. DLBCL gene expression profiles in different ethnic groups have been confirmed to differ between Western and Asian DLBCL patients (33).

The human *NOTCH1* gene is located in the neoplasia-associated region of position 34 of the long arm of chromosome 9 (34). The produced protein may have multiple functions: either an oncogene or a tumor suppressor gene. In this study, we found that patients with the *NOTCH1* mutations had poor PFS and OS, implying an oncogenic role in DLBCL progression. Furthermore, we found *NOTCH1* mutations were negatively associated with complete remission after 6-8 cycles of immunochemotherapy, implying the *NOTCH1* mutation may have predictive potential in the clinical response of DLBCL patients treated with RCHOP chemotherapy. In fact, previous study has reported that *NOTCH1* mutations were associated with lack of benefit of CD20 antibody therapies in chronic lymphocytic leukemia (35). Due to the relatively low mutation frequency, the prognostic value of *NOTCH1* mutations for DLBCL has not been systematically studied previously. In this study, we recruited 161 DLBCL cases in our hospital, among whom patients with the *NOTCH1* mutation had a lower complete response rate than patients without the *NOTCH1* mutation. Similarly, we also enrolled 1562 DLBCL patients treated with R-CHOP from the published DLBCL database to perform meta-analysis, and found a significant association between *NOTCH1* mutations and short PFS and OS, respectively. In addition, we combined personal data from three databases and obtained same results. Thus, *NOTCH1* is conformed to be a potential predictor for DLBCL patients.

DLBCL is a highly heterogeneous tumor type. COO classification and diphenotypic lymphoma (high expression of Bcl2 and c-Myc protein) are commonly known prognostic indicators for clinicians. Non-GCB type DLBCL and double-expression DLBCL both predict poor prognosis. In this study, *NOTCH1* mutation was found to be an independent risk factor for prognosis in our DLBCL patients. This systematic analysis of *NOTCH1* mutation in DLBCL provides data, which support for application of *NOTCH1* mutation detection in clinical diagnosis and treatment, and also provides ideas for finding new therapeutic targets for DLBCL.

However, how the *NOTCH1* mutations affect prognosis and the efficacy of chemotherapeutic drugs remains unclear. It was reported that tumor-infiltrating macrophages (TIMs) are involved in microenvironmental interactions in NOTCH1-mutated patients (36). Monocytes are innate immune cells of the host mononuclear phagocyte system, and its distribution and the transition with macrophages are disrupted in cancer and can affect patient prognosis (37). In fact, peripheral blood monocyte count could reflect the number of local TIMs (38). Our results showed that the *NOTCH1* mutations were significantly associated with low blood monocyte count and high lymphoma/monocyte ratio (LMR). LMR is regarded as a prognostic factor for DLBCL patients (39). The above result supported the fact tumor proliferation promoted by NOTCH1 signals outweighs immune clearance by the host immune system (40). This hypothesis is needed to study in the future.

In conclusion, *NOTCH1* mutations predict a poor progression free survival in DLBCL patients. Targeting of *NOTCH1* mutations could be a potentially effective approach to improve survival of patients.

## Data Availability Statement

The original contributions presented in the study are included in the article/**Supplementary Material**. Further inquiries can be directed to the corresponding authors.

## Ethics Statement

All of the subjects were well-informed about the study and provided written informed consent to participate in the study. The study was approved by the Institutional Review Board of our hospital (No: IIT20210369A).

## Author Contributions

JW, ZL, FY, and JJ designed the research and/or analyzed the data. WLY, YS, JS, and JY carried out the molecular genetic studies, LM and WJY provided clinical data. JW and FY wrote the manuscript. All authors contributed to the article and approved the submitted version.

## Funding

This work is supported by Zhejiang Provincial Natural Science Foundation of China (LY19H080009). The funders had no role in study design, data collection, data analysis, interpretation, writing of this report.

## Conflict of Interest

Authors YS and JY were employed by Nanjing Geneseeq Technology Inc.

The remaining authors declare that the research was conducted in the absence of any commercial or financial relationships that could be construed as a potential conflict of interest.

## Publisher’s Note

All claims expressed in this article are solely those of the authors and do not necessarily represent those of their affiliated organizations, or those of the publisher, the editors and the reviewers. Any product that may be evaluated in this article, or claim that may be made by its manufacturer, is not guaranteed or endorsed by the publisher.
