# Supplementary material for: Clinical Features and Prognostic Significance of NOTCH1 Mutations in Diffuse Large B-Cell Lymphoma
Source: Front Oncol. 2021 Dec 9;11:746577. doi: 10.3389/fonc.2021.746577 (PMC8695434; doi:10.3389/fonc.2021.746577)
Supplement: Supplementary file 1 [file DataSheet_1.pdf]

Supplementary methods

Sample collection and DNA extraction

The NGS tests were performed in a centralized clinical testing center (Nanjing Geneseeq Technology Inc.). Genomic DNA was extracted using the DNeasy Blood& Tissue Kit (Qiagen). All DNA was quantified by Qubit 3.0 using the dsDNA HS Assay Kit (Life Technologies), and the quality was evaluated by a Nanodrop 2000 (Thermo Fisher).

Library preparation and sequencing

Genomic DNA was sheared into fragments (300~350bp) using a Covaris M220 instrument. Fragmented DNA underwent library preparation using KAPA Hyper Prep kit (KAPA Biosystems). In brief, DNA was experienced with end-repairing, A-tailing and adapter ligation, and then was amplified by polymerase chain reaction (PCR) and purified before targeted enrichment. Customized xGen lockdown probes panel (Integrated DNA Technologies) covering 446 predefined cancer-related genes was used to perform hybridization capture. The capture reaction was performed with Dynabeads M-270 (Life Technologies) and the xGen Lockdown Hybridization and Wash kit (Integrated DNA Technologies). Captured libraries were PCR-amplified with KAPA HiFi HotStart ReadyMix (KAPA Biosystems). The purified library was quantified using the KAPA Library Quantification kit (KAPA Biosystems). Finally, the enriched libraries were sequenced on Hiseq 4000 NGS platforms (Illumina) to targeted mean raw coverage depths of at least approximately 700× for tumor genomic DNA.

Data processing and analysis

Sequencing data were demultiplexed using bcl2fastq (v2.19), analyzed by Trimmomatic(Bolger et al., 2014) to remove low-quality (quality<15) or N bases. Then the data were aligned to the hg19 reference human genome with the Burrows-Wheeler Aligner (bwa-mem)(Li and Durbin, 2009) and further processed using the Picard suite (available at: https://broadinstitute.github.io/picard/) and the Genome Analysis Toolkit (GATK)(DePristo et al., 2011). Single-nucleotide variants (SNVs) and insertion/deletions (indels) were called by VarScan2(Koboldt et al., 2012) and HaplotypeCaller/UnifiedGenotyperin GATK.A mutation was called when the mutant allele frequency (MAF) cutoff was ≥ 0.5%, and 4 or more supporting reads with good quality scores from both directions. Common single-nucleotide polymorphisms (SNPs) were removed if they were present at a >1% population frequency in the 1000 Genomes Project or the Exome Aggregation Consortium (ExAC) 65,000-exome database. Called-out variants were further filtered by public databases containing germline mutations, including dbSNP, 1000G, and ExAC, and any variations presented in these databases were removed. The resulting mutation list was further filtered by an in-house list of recurrent artifacts and common SNPs based on approximately 500 whole blood samples (normal pool) from Chinese patients with cancer that were sequenced with the same gene panel at an average depth of 400×. Gene fusions were identified by FACTERA(Newman et al., 2014) and copy number variations (CNVs) were analyzed with ADTEx(Amarasinghe et al., 2013).

References

Amarasinghe, K.C., Li, J., and Halgamuge, S.K. (2013). CoNVEX: copy number variation estimation in exome sequencing data using HMM. *BMC Bioinformatics* 14 Suppl 2**,** S2.

Bolger, A.M., Lohse, M., and Usadel, B. (2014). Trimmomatic: a flexible trimmer for Illumina sequence data. *Bioinformatics* 30**,** 2114-2120.

Depristo, M.A., Banks, E., Poplin, R., Garimella, K.V., Maguire, J.R., Hartl, C., Philippakis, A.A., Del Angel, G., Rivas, M.A., Hanna, M., Mckenna, A., Fennell, T.J., Kernytsky, A.M., Sivachenko, A.Y., Cibulskis, K., Gabriel, S.B., Altshuler, D., and Daly, M.J. (2011). A framework for variation discovery and genotyping using next-generation DNA sequencing data. *Nat Genet* 43**,** 491-498.

Koboldt, D.C., Zhang, Q., Larson, D.E., Shen, D., Mclellan, M.D., Lin, L., Miller, C.A., Mardis, E.R., Ding, L., and Wilson, R.K. (2012). VarScan 2: somatic mutation and copy number alteration discovery in cancer by exome sequencing. *Genome Res* 22**,** 568-576.

Li, H., and Durbin, R. (2009). Fast and accurate short read alignment with Burrows-Wheeler transform. *Bioinformatics* 25**,** 1754-1760.

Newman, A.M., Bratman, S.V., Stehr, H., Lee, L.J., Liu, C.L., Diehn, M., and Alizadeh, A.A. (2014). FACTERA: a practical method for the discovery of genomic rearrangements at breakpoint resolution. *Bioinformatics* 30**,** 3390-3393.

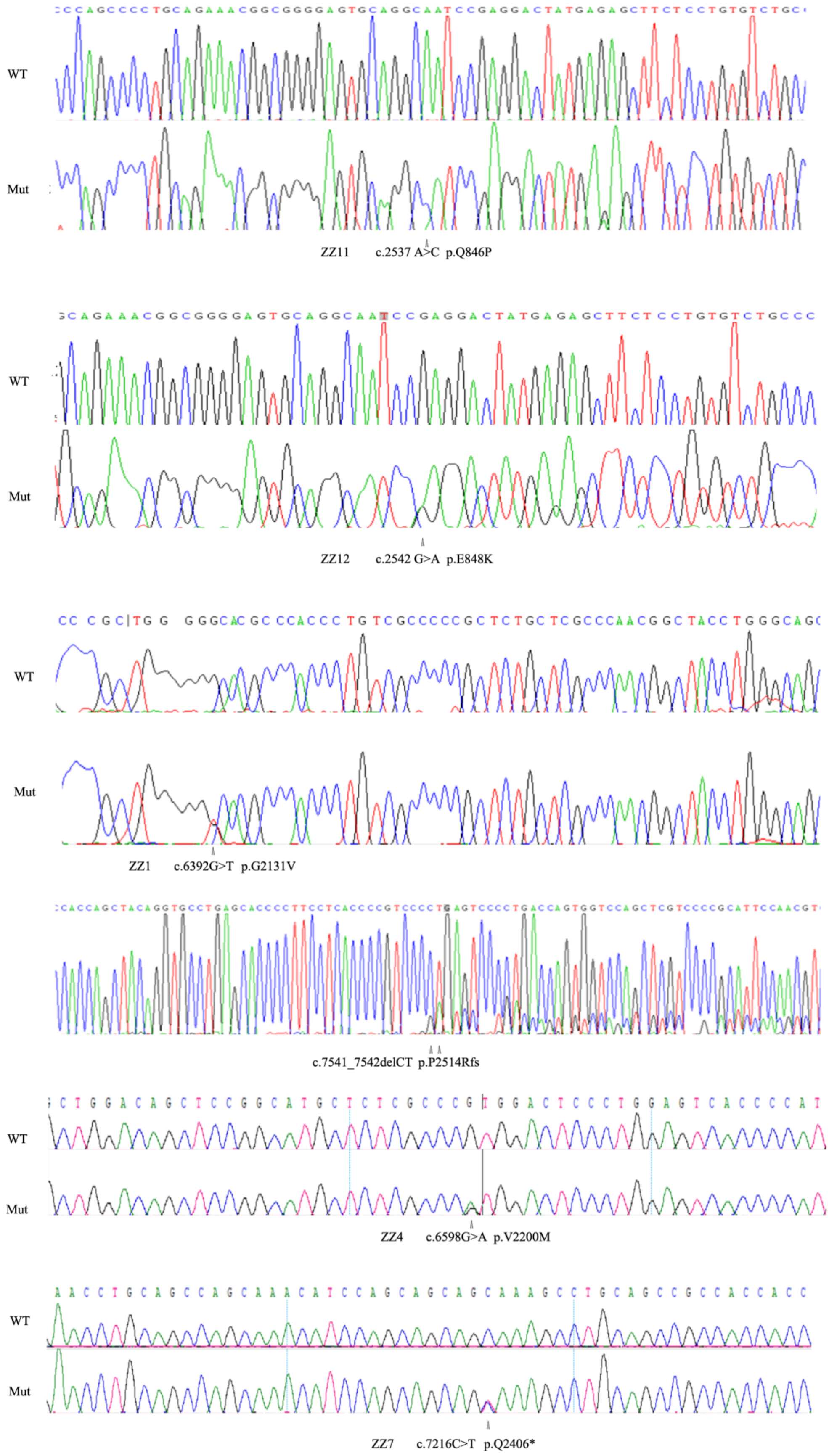

Figure S1. *NOTCH1* mutations were validated by Sanger sequencing.

Substitution	Preservation time	Protein function
T1997M	307	Possibly damaging
G2131V	797	Possibly damaging
E334K	797	Possibly damaging
V2200M	797	Possibly damaging
R2549C	307	Possibly damaging
Q846P	307	Possibly damaging
E848K	307	Possibly damaging
P3R	307	Possibly damaging
P2334L	307	Possibly damaging
E1305K	307	Possibly damaging
R207C	307	Possibly damaging

Figure S2. The protein function of missense mutations estimated by cSNP tool(http://pantherdb.org/tools/csnpScore.do). Estimates the likelihood of a particular nonsynonymous (amino-acid changing) coding SNP to cause a functional impact on the protein. It calculates the length of time (in millions of years) a given amino acid has been preserved in the lineage leading to the protein of interest. The longer a position has been preserved, the more likely that it will have a deleterious effect.

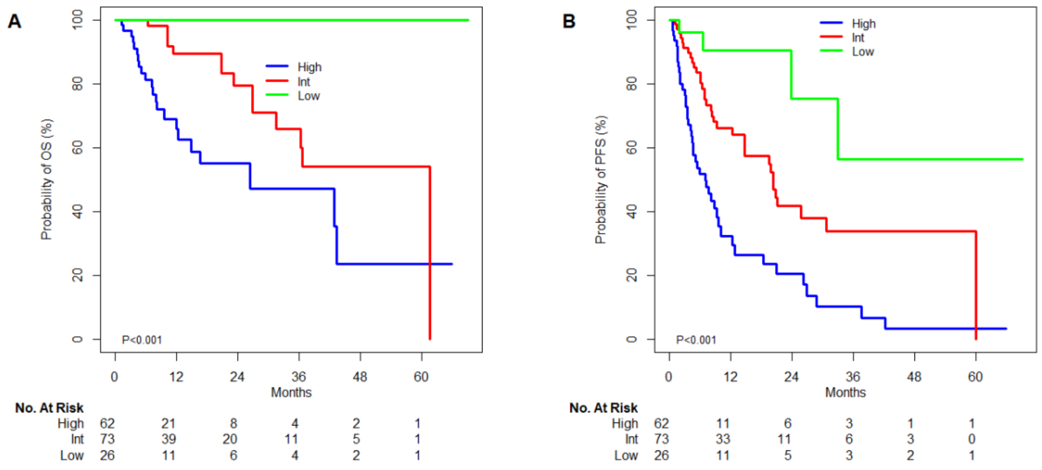

Figure S3. Survival curves of OS (A) and PFS (B) by IPI levels.

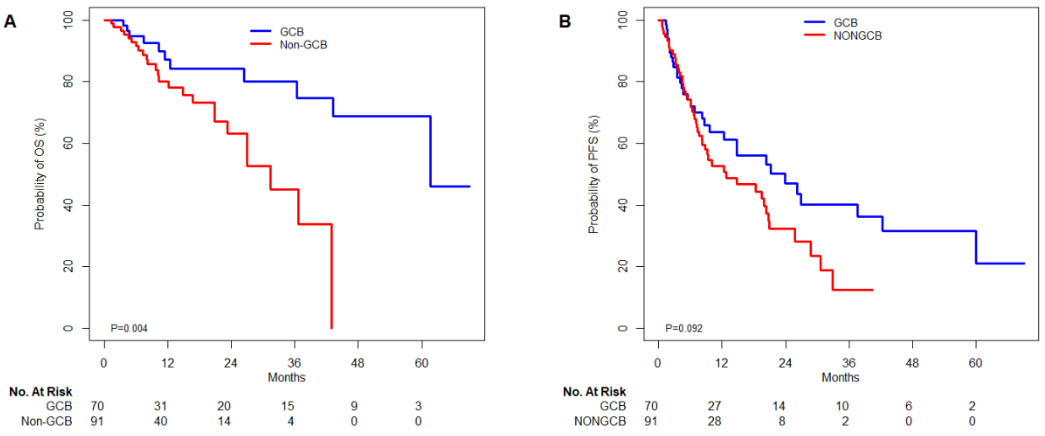

Figure S4. Survival curves of OS (A) and PFS (B) by COO classifications.

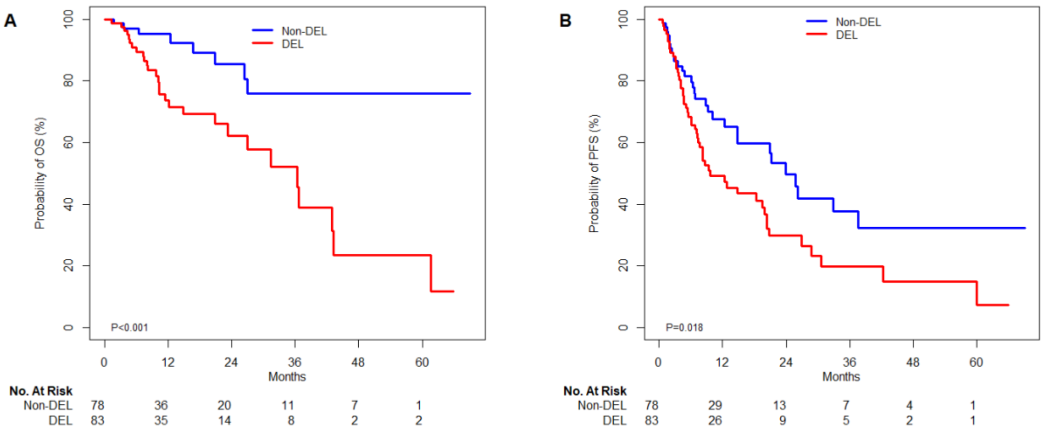

Figure S5. Survival curves of OS (A) and PFS (B) by distinct DEL subgroups.

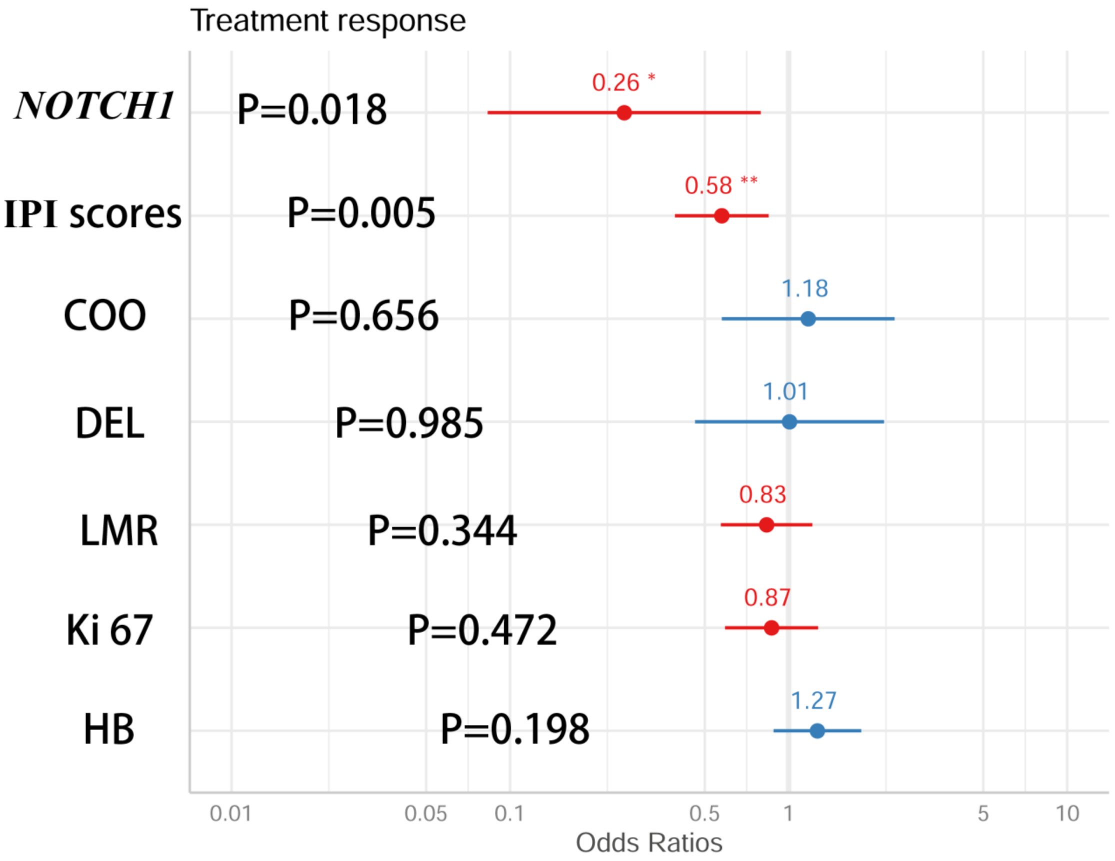

Figure S6. Multivariate logistic regression in DLBCL patients. "*" represents p value less than 0.05, "**" represents P<0.01. Parameters with blue bars indicate these parameters are positively associated with CR and red bars were negatively associated with CR.

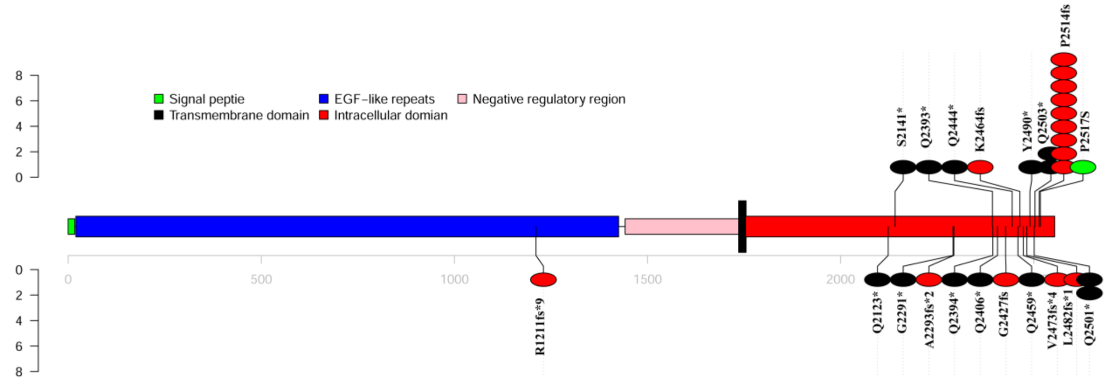

Figure S7. Mutation maps of NOTCH1 protein reanalyzing from the previous studies. The x-axis reports the amino acid number, the y-axis reports the number of the mutations. The circles are colored with respect to the corresponding mutation types: “black” representing non-sense mutations, “red” equaling to frameshift mutations, “green” representing missense mutations.

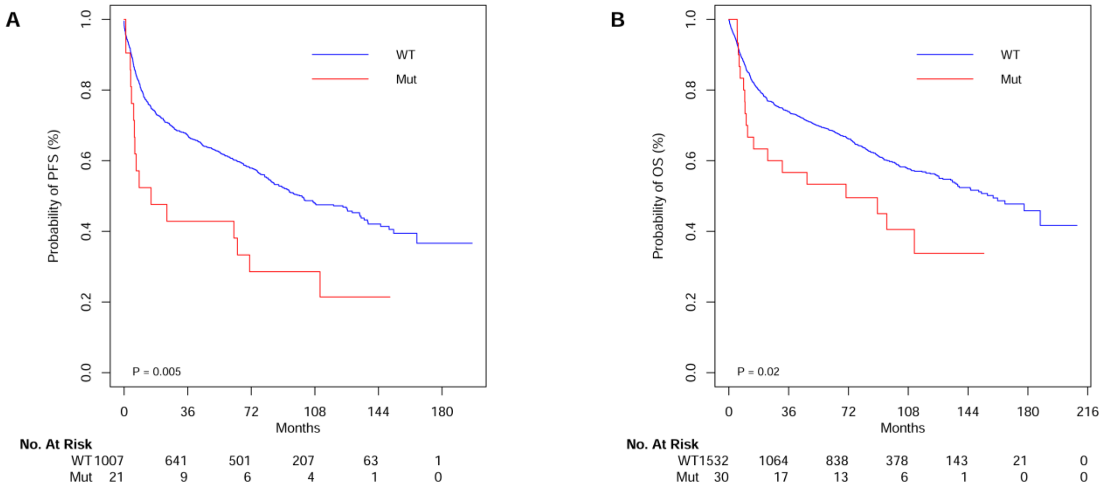

Figure S8. Survival curves of PFS (A) and OS (B) in the combined individual data of the three western cohorts of DLBCL patients. With respect to survival analysis, we included the patients with the available information of clinical outcomes and treated with R-CHOP regiments. For the PFS and OS analysis, there are 578 and 630 cases without *NOTCH1* mutations and 18 and 16 with *NOTCH1* mutations from the Stuart E. Lacy’s study; zero and 7 patients with *NOTCH1* mutations and 357 and 830 without *NOTCH1* mutations from the study of George W. Wright et al; 72 without *NOTCH1* mutations and 5 with *NOTCH1* mutations from the study of Lucía Pedrosa et al.

Table S1. The primers of *NOTCH1* mutations.

NOTCH1-2F	ACCAATACAACCCTCTGCGG
NOTCH1-2R	ACTTGAAGGCCTCCGGAATG
NOTCH1-2F	GTGCACGTGGTTCCCACATA
NOTCH1-2R	CCAGGAGTAGGCTTCCGTGA
NOTCH1-2F	GGCAGGATGTCAACGAGTGT
NOTCH1-2R	CCTACGTACAACACGGGGAT
NOTCH1-2F	CTGCTGGACGAGTACAAC
NOTCH1-2R	GTCTGACAGGTAGCCATG
NOTCH1-2F	ctaggaccacagtgaggat
NOTCH1-2R	GGCGTGTGAGTTGATGAG
NOTCH1-2F	AACGAGTGCCTGTCCAAT
NOTCH1-2R	agtctacttcctgctccatt

Table S2. The detailed information about *NOTCH1* mutations.

Number	Variant_Classification	Variant_Type	Base_Change	Protein_Change	VAF	Sanger sequencing
ZZ1	Missense_Mutation	SNV(Type 1)	c.1000G>A	p.E334K	0.43	NA
ZZ1	Missense_Mutation	SNV(Type 1)	c.6392G>T	p.G2131V	0.56	Positive
ZZ2	Frame_Shift_Del	DEL(Type 1)	c.7541_7542delCT	p.P2514Rfs*4	0.79	NA
ZZ3	Missense_Mutation	SNV(Type 1)	c.2510C>T	p.P837L	0.07	Negative
ZZ4	Missense_Mutation	SNV(Type 1)	c.6598G>A	p.V2200M	0.65	Positive
ZZ5	Missense_Mutation	SNV(Type 1)	c.3913G>A	p.E1305K	0.11	NA
ZZ6	Missense_Mutation	SNV(Type 1)	c.5990C>T	p.T1997M	0.29	NA
ZZ7	non-sense mutations	SNV (Type 2)	c.7216C>T	p.Q2406*	0.54	Positive
ZZ8	non-sense mutations	SNV (Type 2)	c.6511A>T	p.K2171*	0.12	NA
ZZ9	Frame_Shift_Del	DEL(Type 1)	c.7541_7542delCT	p.P2514Rfs*4	0.18	NA
ZZ10	Missense_Mutation	SNV(Type 1)	c.7645C>T	p.R2549C	0.52	NA
ZZ11	Missense_Mutation	SNV(Type 1)	c.2537A>C	p.Q846P	0.48	Positive
ZZ12	Missense_Mutation	SNV(Type 1)	c.2542G>A	p.E848K	0.5	Positive
ZZ13	Frame_Shift_Del	DEL (Type 2)	c.10_23delCTCCTGGCGCCCCT	p.L4Afs*21	0.74	NA
ZZ13	Frame_Shift_Del	DEL (Type 2)	c.8_23delCGCTCCTGGCGCCCCTinsGG	p.P3Rfs*22	0.74	NA
ZZ14	Missense_Mutation	SNV(Type 1)	c.619C>T	p.R207C	0.39	Negative
ZZ15	Missense_Mutation	SNV(Type 1)	c.7001C>T	p.P2334L	0.48	NA
ZZ16	Frame_Shift_Del	DEL(Type 1)	c.7541_7542delCT	p.P2514Rfs*4	0.76	Positive
ZZ17	Splice_Site	SNV (Type 2)	c.865+3G>A	.	0.33	NA

We named mutations potentially affecting the NOCH1 activity as type 1 group and mutations probably leading to the absence of protein expression as type 2 group. Due to no samples stored in our biobank, we examined 8 out of 16 mutated sites in our patients.

Table S3. Clinical characteristics of patients with different types of mutations

Characteristics	Type 1 group	Type 2 group	P values
Number	13	4	
Male, sex,n(%)	10(76.9)	1(25.0)	0.099
Age, Median(IQR)1	62.00[54.00,66.00]	48.00[32.75,65.00]	0.395
Age(>60yrs), n(%)	7(53.8)	2(50.0)	1
ECOG PS(>=2), n(%)2	8(61.5)	3(75.0)	1
LDH, IU/L, Median(IQR)	301.00[189.00,456.00]	236.50[229.50,389.00]	1
Stage III-IV	13(100.0)	4(100.0)	1
Extranodal disease, n(%)	7(53.8)	3(75.0)	0.603
IPI, n(%)3			0.129
Low	0(0.0)	0(0.0)	
Intermediate-low	4(30.8)	0(0.0)	
Intermediate-high	2(15.4)	3(75.0)	
High	7(53.8)	1(25.0)	
Non-GCB, n(%)4	7(53.8)	2(50.0)	1
CD10 positive	3(23.1)	1(25.0)	1
Bcl6 positive	13(100.0)	4(100.0)	1
Mum1 positive	10(76.9)	3(75.0)	1
DEL, n(%)5	6(46.2)	3(75.0)	0.576
MYC positive	8(66.7)	4(100.0)	0.516
BCL2 positive	11(84.6)	3(75.0)	1
Ki-67, Median(IQR)	80.00[62.00,80.00]	57.50[43.75,75.00]	0.563
DH/TH, n(%)6	0(0.0)	0(0.0)	1
BCL6 fusion,n(%)	1(7.7)	0(0.0)	1
MYC translocation,n(%)	1(7.7)	0(0.0)	1
BCL2 fusion,n(%)	1(7.7)	0(0.0)	1
B symptoms,n(%)7	2(15.4)	2(50.0)	0.219
WBC(median[IQR])	6.00[3.52,7.50]	5.15[4.32,6.31]	0.61
HB(median[IQR])	139.00[130.00,142.00]	124.50[114.00,136.75]	0.308
PLT(median[IQR])	212.00[146.00,242.00]	213.00[150.50,242.75]	1
LMR(median[IQR])	2.61[1.71,5.93]	4.86[3.88,6.54]	0.258
N(median[IQR])	3.40[2.71,4.50]	3.25[2.25,4.20]	0.734
L(median[IQR])	1.14[0.74,2.20]	1.80[1.17,2.44]	0.461
M(median[IQR])	0.46[0.29,0.55]	0.29[0.26,0.36]	0.234
Response to treatment, n(%)9			1
CR	4(30.8)	2(50.0)	
PD	5(38.5)	1(25.0)	
PR	4(30.8)	1(25.0)	

[1]IQR, interquartile range; [2] ECOG PS, Eastern Cooperative Oncology Group performance status;

[3]IPI, International Prognostic Index; [4] Non-GCB, Non-germinal center B-cell-like lymphoma; [5]DEL, double expresser lymphoma; [6]B symptoms refer to systemic symptoms of fever, night sweats, and weight loss; [7]CR, complete remission. WBC, white blood cell counts; HB, hemoglobin; PLT, platelet count; LMR, lymphoma-to-monocyte ratio.

Table S4. Characteristics of DLBCL patients with and without *NOTCH1* mutations

Characteristics	WT	Mutant	P values
Number	144	17	
Male, sex, n(%)	83(57.6)	11(64.7)	0.62
Age, Median(IQR)1	58.00[46.75,67.00]	62.00[54.00,66.00]	0.81
Age (>60yrs), n(%)	66(45.8)	9(52.9)	0.62
ECOG PS (>=2), n(%)2	83(57.6)	11(64.7)	0.62
LDH, IU/L, Median(IQR)	303.00[217.75,514.00]	282.00[195.00,456.00]	0.55
Stage III-IV	97(67.4)	17(100.0)	0.003
Extranodal disease, n(%)	90(62.5)	10(58.8)	0.8
IPI, n(%)3			0.23
Low	26(18.1)	0(0.0)	
Intermediate-low	25(17.4)	4(23.5)	
Intermediate-high	39(27.1)	5(29.4)	
High	54(37.5)	8(47.1)	
Non-GCB, n(%)4	82(56.9)	9(52.9)	0.80
CD10 positive	47(32.6)	4(23.5)	0.59
Bcl6 positive	126(90.0)	17(100.0)	0.37
Mum1 positive	107(74.8)	13(76.5)	1
DEL, n(%)5	74(51.4)	9(52.9)	1
MYC positive	86(68.8)	12(75.0)	0.78
BCL2 positive	113(82.5)	14(82.4)	1
Ki-67, Median(IQR)	80.00[66.50,90.00]	70.00[45.00,80.00]	0.32
B symptoms, n(%)6	34(23.8)	4(23.5)	1
WBC (median [IQR])	5.95 [4.50, 8.03]	5.80 [3.52, 7.50]	0.67
HB (median [IQR])	122.00 [106.00, 134.00]	136.00 [125.00, 142.00]	0.006
PLT (median [IQR])	213.00 [144.75, 258.50]	212.00 [146.00, 242.00]	0.54
LMR (median [IQR])	1.99[1.10,3.44]	3.67[2.07,5.93]	0.02
N (median [IQR])	3.92[2.60,5.43]	3.40[2.40,4.49]	0.21
L (median [IQR])	1.10[0.75,1.54]	1.30[0.76,2.30]	0.35
M (median [IQR])	0.55[0.37,0.79]	0.38[0.28,0.52]	0.031
CR, n(%)7	94(65.3)	6(35.3)	0.028

[1]IQR, interquartile range; [2] ECOG PS, Eastern Cooperative Oncology Group performance status;

[3]IPI, International Prognostic Index; [4] Non-GCB, Non-germinal center B-cell-like lymphoma; [5]DEL, double expresser lymphoma; [6]B symptoms refer to systemic symptoms of fever, night sweats, and weight loss; [7]CR, complete remission. WBC, white blood cell counts; HB, hemoglobin; PLT, platelet count; LMR,

lymphoma-to-monocyte ratio.

Table S5. Univariate analysis of PFS and OS in DLBCL patients

Variables	Progression free survival		Overall survival	
	P values	HR(95%CI)	P values	HR(95%CI)
Mutant vs. WT	0.016	1.996(1.135,3.508)	0.04	2.225(1.039,4.764)
IPI scores	<0.001	1.637(1.367,1.959)	<0.001	1.815(1.365,2.412)
Non-GCB vs. GCB	0.097	1.479(0.932,2.346)	0.01	2.975(1.336,6.628)
DEL vs Non-DEL	0.02	1.711(1.09,2.687)	<0.001	3.523(1.596,7.774)
LMR	0.54	1.146(0.741,1.771)	0.07	0.533(0.269,1.054)
HB	0.087	0.991(0.982,1.001)	0.05	0.986(0.972,1)
KI-67	0.138	1.008(0.998,1.018)	0.05	1.018(1,1.035)

IPI, international prognostic index; GCB, Germinal center B-cell-like lymphoma; DEL, double expresser lymphoma.
